# Aquaporins in Sensory and Pain Transmission

**DOI:** 10.2174/157015910791233187

**Published:** 2010-06

**Authors:** Elisa Borsani

**Affiliations:** Division of Human Anatomy, Department of Biomedical Sciences and Biotecnologies, University of Brescia, V.le Europa 11, 25123 Brescia, Italy

**Keywords:** Aquaporins, nervous system, sensory transmission, pain transmission.

## Abstract

Recent data suggest a possible involvement of Aquaporins (AQPs) in pain transmission. AQPs are small membrane channel proteins involved in osmoregulation and, to date, AQP1, AQP2, AQP3, AQP4, AQP5, AQP8 and AQP9 have been found in the nervous system. Nevertheless only AQP1, AQP2 and AQP4 seem to be involved in nociception.

In this review, direct and indirect evidences of the role of AQPs in pain processing will be reported.

## 1. AQUAPORINS LOCALIZATION IN SENSORY NOCICEPTIVE STRUCTURES 

AQP1 is expressed in peripheral nerve fibers that projects to the dorsal horn, which is involved in pain sensation [35]. In spinal cord AQP1 was observed in small afferent sensory nerve fibers [35]; specifically mRNA for AQP1 is concentrated in a subset of neuronal cell bodies in the dorsal root ganglia (DRG), but not in spinal cord neurons [40]. Moreover, AQP1 has been found in small diameter sensory neurons in dorsal root, trigeminal and nodose ganglia, but studies of a possible role for AQP1 in pain sensation have yielded conflicting results [35, 40]. Taken together, these data indicate that the spinal cord AQP1 derives exclusively from primary sensory neurons targeting neurons in the superficial dorsal horn. Osmotically induced swelling of the spinal cord was reduced in AQP1 knock-out mice [42] and markedly impaired pain sensation was demonstrated in response to thermal (tail flick test) and chemical (capsaicin injection) stimuli [34], suggesting a role for AQP1 in neural signal transduction, and in rapid water recycling. 

AQP2 expression is present in spinal cord, in subcortical white matter and hippocampus [28]. In addition, weak AQP2 immunoreactivity is found in peripheral nerves [28] and in dorsal root and trigeminal ganglia neurons where the immunostaining is in the cytoplasm of each neuronal class [6, 7].

AQP4 is the predominant subtype present in the brain [47] and spinal cord [33]. The most abundant site of AQP4 expression in the brain and spinal cord is the perivascular glial processes [32, 33, 44]. In fact, AQP4 is a glial [43] membrane channel involved in cellular osmotic brain regions, including the locus coeruleus and the ventral tegmental area (VTA) or the extended amygdala, nucleus accumbens, and prefrontal cortex [4] and knock-out of the Aqp4**gene modifies cocaine-related behaviours [24]. Additionally, AQP4 is localized in the Purkinje layer of the cerebellum, as well as in the supraoptic and paraventricular nuclei of the hypothalamus [32]. AQP4 is also expressed in ependymal cells, but is absent from neurons, oligodendrocytes, and microglia [32]. Water transport via AQP4 is essential for normal neuronal activity. Binder and co-workers [5] have shown that seizure susceptivity was increased in AQP4 deleted mice suggesting that glial water channels may modulate brain excitability and the initiation and generalization of seizure activity.

The presence of AQP3, AQP5, and AQP8, AQP9 was reported in central nervous system (CNS) [39], but their physiological role has not been fully analyzed.

## 2. EVIDENCES OF AQUAPORINS INVOLVEMENT IN PAIN TRANSMISSION

### 2.1. AQP1

The role of AQP1 in pain processing is debated.

Three gene clusters containing 11 genes has been identified as a potential pool of candidate genes related to somatosensation in cranial structures such as the face, oral cavity and pharynx [27]; among them we find AQP1. The results suggested that the AQP1-positive cells are probably somatosensory neurons in two cranial sensory ganglia, the trigeminal and petrosal ganglia. The expression profiles and probable physiological functions of the 6 genes such as trkA, NaN and galanin suggest their direct involvement in specific somatosensory functions such as nociception. AQP1 has been identified in those ganglia and is expressed in small to medium size neurons with a diameter under 30 um [27].

The glial cells in the peripheral nervous system (PNS) express specifically AQP1 protein, while the astrocytes, the equivalent cells in the CNS, express AQP4 and AQP9 [12]. This finding suggests that members of the AQP family are differentially expressed in the PNS *versus* the CNS [12]. Glial cells have extensive roles in regulating extracellular concentrations of ions, metabolites, and neurotransmitters [41]. Increasing evidence indicates that glial cells function to coordinate the differentiation, metabolism, and excitability of neurons, to modulate synaptic transmission, and to integrate signals from neurons and other glial cells [3, 11]. AQP1 expression in glial cells of the PNS suggests involvement of channel-mediated water transport mechanisms in peripheral neuronal activity by regulating water homeostasis in the nerve plexuses and bundles, which warrants further investigations [12]. 

AQP1 has also an important role in synaptic vesicle swelling that is critical for secretion. Recent study identifies AQP1, but also AQP6, associated with synaptic vesicles [17].

In a communication of Oshio and co-workers [34] and after in a ‘commentary work’ of Verkmann [45], the information that mice lacking AQP1 reduced nociception in response to thermal stimuli and capsaisin was reported. After, the first complete work which attributed to AQP1 a role in pain transmission was published by Oshio and co-workers [35]. They observed that AQP1 is expressed in small afferent sensory nerve fibers in both the CNS and the PNS and that AQP1 deletion is associated with reduced behavioral response to thermal and capsaicin chemical stimuli. These results suggested a functional role for AQP1 in nociceptive neurons [35]. The behavioral analysis demonstrated that AQP1 appears to contribute to the processing of two principal types of acute pain: thermal and chemical (capsaicin). It is of particular interest that the pain phenotype of AQP1 knock-out mice is similar to TRPV1 (transient receptor potential vanilloid 1) deficient mice which have normal responses to noxious mechanical stimuli and the formalin test [8].

For acute thermal pain, both the AQP1 and TRPV1 null mice have reduced heat-evoked responses. AQP1 KO mice also have a significantly attenuated response to capsaicin, although not to the same degree as TRPV1 null mice where the response to capsaicin is completely lost. It is intriguing that dorsal root stimulation results in activity-dependent water flux specifically in the superficial dorsal horn of the spinal cord where AQP1 is expressed. Given the high surface area-to-volume ratio of small diameter C-fibers as compared to A-fibers, it has been speculated that AQP1 may be necessary to dissipate osmotic gradients associated with rapid ion transport in these neurons. A similar mechanism has been proposed for AQP4 in facilitating K^+^ ion flux in astroglia, Muller cells, and the supportive epithelial cells in the cochlea [25, 2]. Thus, it is possible that AQP1 may play a similar role in nociceptive neuronal ion homeostasis. 

A number of previous studies have provided indirect evidence of a contribution of osmosis in the pain pathway. Over 30 years ago, Hitchcock and others reported that intrathecal injections of cold saline in patients with intractable pain led to immediate relief [14]. This effect was attributed to the osmolality and temperature of the solution. In other studies, Jewett and co-workers [18] and King and co-workers [20] used an *in vitro* preparation of dorsal roots from either monkeys or cats to examine the effects of saline of various osmolalities on the firing of A and C-fibers. Interestingly, they found that distilled water or hypotonic saline resulted in differential blockade of C-fibers, with little or no effect on A-fibers. It was proposed that differences in water flux underlie the preferential conduction block of C-fibers. Although anatomical differences between A and C-fibers were offered as a possible explanation, they suggested that the differential expression of AQP1 in C-fibers may account for the results and provide a molecular basis for osmosis in the pain pathway. 

Other recent evidences support the hypothesis of a role of AQP1 in pain state [31]. Rat contusion spinal cord injury (SCI) induced persistent and significant four- to eightfold increases in AQP1 levels at the site of injury (T10) persisting up to 11 months post-contusion. Interestingly; AQP1 levels were not affected by long-lasting hypertonicity that significantly increased astrocytic AQP4, suggesting that the primary role of AQP1 is not regulating isotonicity in spinal cords. They proposed possible novel roles for AQP1 in the injured spinal cords in neuronal and astrocytic swelling, as AQP1 was increased in all surviving neurons and reactive astrocytes after SCI and in the development of the neuropathic pain after SCI. In fact, they have shown that decreased AQP1 in melatonin-treated SCI rats correlated with decreased AQP1 immunolabeling in the dorsal horns sensory afferents and with significantly decreased mechanical allodynia, suggesting a possible link between AQP1 and chronic neuropathic pain after SCI [31].

Moreover, AQP1 transports nitric oxide (NO) across cell membranes [13] that is NO transport is facilitated by a specific carrier such as AQP1. In fact, NO permeability of CHO-K1 cells (cell line from hamster ovary) increases with water permeability, which is a marker of AQP1 expression. This is the first direct evidence that an integral membrane transport protein facilitates diffusion of NO. Alterations in AQP-mediated NO transport may be an alternate explanation for many diseases and could be influence the neuromodulation, in which NO is primarily implicated [26].

A completely opposed point of view was primarily proposed by Shields and co-workers [40]. Despite the heavy expression of AQP1 in nociceptors, and in marked disagreement with recently published data [35], they found absolutely no deficits in nociceptive processing in AQP1 mutant mice. It is important to observe that although AQP1 labeling in the dorsal horn changes after sciatic nerve transection, a manipulation that results in an altered pain state producing changes in nociceptor physiology, neither electrophysiological nor a very complete behavioural analysis uncovered differences in nociceptive processing between mice lacking AQP1 and wild-type mice. The reason for this discrepancy is not clear but is unlikely to be related to testing methods or to mouse background; these factors did not differ between the studies. Although each thermal pain assay measures a slightly different parameter, it is significant that in diverse tests of thermal pain sensibility (hindpaw radiant heat, hot plate, tail flick, thermal allodynia after nerve injury or CFA (complete Freund’s adjuvant) injection, and electrophysiological responses to heat stimuli) it has been found that AQP1 knock-out mice process noxious heat stimuli normally. 

Supporting these findings there are observations in humans; all AQP1 null humans studied to date have been reported to be free from neurological dysfunction [37], and there is no indication that they have altered pain sensation. Moreover, in a recent work in a mouse model of formalin trigeminal acute inflammatory pain, there are no alterations in AQP1 expression in trigeminal ganglia as reported in Fig. (**1**) [6]. In addition, another recent work based on a neuropathic rat model of chronic constriction injury (CCI) of sciatic nerve, reported no alteration of AQP1 expression both in dorsal root ganglia and in spinal cord [7].

### 2.2. AQP2

Direct experimental evidences of AQP2 involvement in pain transmission have been published very recently. AQP2 expression undergoes interestingly changes in trigeminal ganglia during acute inflammation as reported in Fig. (**2**) [6]. In the control animals the AQP2 immunostaining showed general staining in the cytoplasm of each neuronal class, the staining was stronger in the medium- and large-sized neurons than in the small one while neuronal membranes were weakly positive; Schwann cells were negative and satellite cells were weakly positive. In formalin treated animals, AQP2 increased from a quantitative point of view and a redistribution in trigeminal ganglia cells was observed. AQP2 expression increased in the cytoplasm and neuronal membrane of small-sized neurons, while the AQP2 immunostaining decreased in the cytoplasm of medium/large sized neurons with a strong increase in the neuronal membrane. Therefore, neuronal membranes had heavy staining in all neuronal classes, the Schwann cells and the satellite cells were highly positive compared to the control group. These observations lead to consider the redistribution of AQP present in the cytoplasm in the membrane compartment for an adaptation in nociceptive condition. 

Moreover, in the neuropathic rat model of chronic constriction injury (CCI) of sciatic nerve was assessed the alteration of AQP2 expression in DRG [7]. CCI treatment induced a significant increase of AQP2 expression restricted to DRG and not in spinal cord. In DRG of CCI treated animals, Buffoli and co-workers [7] observed immunopositive neurons, scattered throughout the ganglia, showing a variable degree of staining; in particular, the most intensely stained cells were small diameter sized, therefore in the neurons that co-label for markers of nociceptors as reported in Fig. (**3**). On the contrary, its expression was very weak in DRG of naïve and sham-operated rats and negative in spinal cord and sciatic nerve of all animals. Considering that the staining was localized mainly in the cytoplasm of small diameter sized neurons, these observations lead to consider the distribution of AQPs in the cytoplasm with respect to membrane compartment for an adaptation in chronic nociceptive condition in acute pain status. These could be related with the results obtained in trigeminal ganglia during acute inflammation, in which a redistribution of AQP2 in the neuronal membrane was observed [6]. Although there are evidences that AQP2 mRNA is also present in human spinal cord [28], these results showed AQP2 staining only in DRG and not in the lumbar spinal cord. Moreover, CCI treatment did not induce AQP2 expression at the CCI injury site but in some small vessels of DRG. This could be due to the development of peripheral edema associated with this pain model [23].

The exact mechanism by which AQP2 is enhanced is not clear. It is known that this protein translocates in response to arginine vasopressin (AVP) also in DRG [15, 19]. In particular, there are evidences that AVP induced hyperpolarization in the membrane of most DRG neurons which might be caused by K(+) outflow mediated by V1 receptor and that the membrane conductance of the DRG neurons increased following AVP application [15, 16]. 

### 2.3. AQP4

AQP4 is a protein implicated in neurotransmission [45], also considering the recent important localization in sensory neurons [1], but only two experiments [22, 30] have been performed with the aim to link it to pain perception, and only one has had some positive results in this direction [30]. 

Nesic and co-workers [30] analysed AQP4 expression in central neuropathic pain (CNP) syndrome in a rat model of SCI, because it severely affects the quality of life of SCI patients. AQP4 is an astrocytic water channel protein that is typically up-regulated in activated astrocytes. DNA microarray analysis showed significantly increased expression of a number of genes associated with inflammation and astrocytic activation in the spinal cords of rats that developed CNP. mRNA levels of glial fibrilary acidic protein (GFAP) and AQP4 significantly increased in CNP spinal cords of rats. A representative western blot shows low AQP4 expression in uninjured samples, and highly increased AQP4 protein levels in the CNP spinal cords, consistent with GFAP expression changes. Interestingly, GFAP and AQP4 are not only increased at the site of injury at 4 weeks after SCI, but also in the spinal segments away from the site of injury; in lumbar (L4/L5 combined) and in cervical (C7/C8 combined) segments in the spinal cords of rats with different level of CNP.

Opposing results were obtained by Lacroix and co-workers [22]. They aimed to identify significative alteration of AQP4 in a rat model of lumbar radiculopathy (L5 nerve root ligation) and neuropathy (L5 spinal nerve transection). The authors measured mechanical allodynia followed by analysis of global gene expression in the lumbar spinal cord at two time points (7 days and 14 days). Finally, they did not observe significant difference in AQP4 expression between any of the treatment groups.

These contrasting results lead our attention to the complexity of AQP function probably linked to the experimental model.

Other recent experiments link AQP4 to sensory transduction and could induce to correlate the results to a possible role of this water channel to pain transmission, even if other direct correlation was never directly done. 

Evidences showed that AQP4 plays an important role in functions of astrocyte, such as astroglial migration, K^+^ buffering, and neuronal activities [5, 36, 38]. In astrocytes, AQP4 coexists extensively with inward rectifying K^+^ channel 4.1 (Kir 4.1), glial glutamate transporter 1 (GLT1) and glutamate/aspartate transporter (GLAST), the major transporters responsible for clearance of extracellular glutamate [29, 32, 46, 49]. Additionally, ultrastructural examinations showed that astrocytes AQP4-positive processes surround certain synapses (excitatory and inhibitor synapses) [32, 46]. These indicate that AQP4 might modulate excitatory synaptic transmission. Glutamate is the principle excitatory neurotransmitter in the CNS, which participates in many physiological functions and pathological processes including nociception which suggests that AQP4 could modulate K^+^ and glutamate homeostasis. 

In brain, seizure susceptibility in response to the convulsant (GABA antagonist) pentylenetetrazol was remarkably increased in AQP4 null mice [5]. Some investigations have demonstrated that the lack of AQP4 expressions in mice is paralleled by sex- and region-specific alterations in neurotransmission [10]. Therefore, AQP4 may serve as a modulator of astroglial function including regulating neurotransmitter release in mammalian brain. AQP4 deficiency influences K^+^-stimulated releases of striatal neurotransmitters [9]. 

AQP4 may play a role in regulating extracellular cocaine-induced dopamine and glutamate release in the brain reward center, and in turn AQP4 deletion may attenuate cocaine reinforcement and dependence [24]. 

Wu and co-workers [48] presumed that there might be a relationship between AQP4 and opioid analgesia, tolerance and dependence processes through glial glutamate transporters and glutamatergic neurotransmission. A reduction in basal GLT1 expression in cerebrum was observed in AQP4 knock-out mice. Compared with wild-type mice, the basal GLT1 expression in cerebrum was decreased by 23.1% and 26.5% in male and female AQP4 knock-out mice, respectively. Moreover, AQP4 deficiency potentiated morphine analgesia, attenuated morphine tolerance and physical dependence; the suppression of down-regulation of GLT1 expression might mediate the attenuation of AQP4 deficiency to morphine tolerance and dependence [48].

Recently, some evidences suggest that neural activity-dependent astrocyte swelling is likely to be mediated by water influx through AQP4 water channels [21].

## CONCLUSIONS

The role of AQPs in pain processing is today in open debate. It is true that a lot of direct and indirect evidences strongly lead to hypothize an active role in pain modulation, but important data do not agree with this. So, although there is not in literature a unidirectional way on this point, the inter-relationship between nervous system/neurotransmission and AQPs is otherwise an undisputed embraced new concept.

## Figures and Tables

**Fig. (1):**
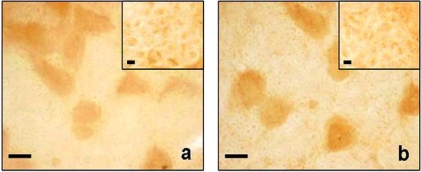
AQP1 immunostaining of trigeminal ganglia of control (**a**) and formalin (**b**) animals. The main microphotograph represent neurons while the inset-axons. Bar: 20um for (**a**) and (**b**); 5um for the insets (previously published in Borsani E *et al*., 2009 - Elsevier).

**Fig. (2):**
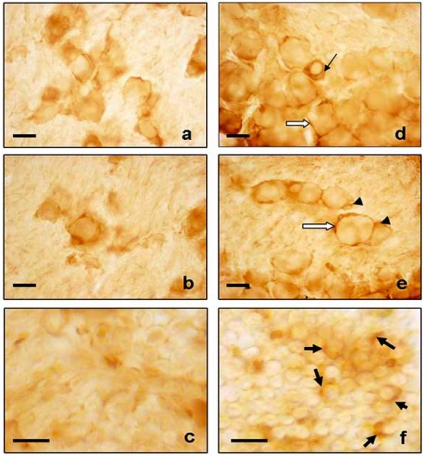
AQP2 immunostaining of trigeminal ganglia of control (**a**-**c**) and formalin (**d**-**f**) animals. In formalin treated animals, a strong immunopositivity was found in cytoplasm of small-sized neurons (black thin arrow), neuronal membranes (white arrow), Schwann cells (black thick arrow) and satellite cells (head arrow). Bar: 20um (**a**, **b**, **d**, **e**); 10um (**c**, **f**) (previously published in Borsani E *et al*., 2009 - Elsevier).

**Fig. (3):**
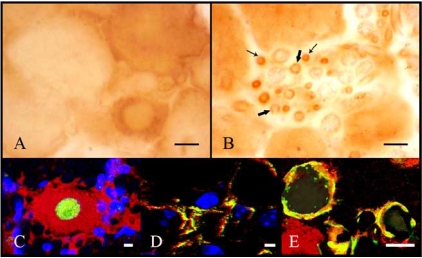
Immuohistochemical analysis of AQP2 expression in the DRG of CCI rats. (**A**) neuronal immunostaining was mainly intracytoplasmatic, whereas the nucleus appeared unstained. (**B**) Schwann cells were weakly stained (thick arrows) and several small vessels were strongly immunopositive (thin arrows). Double immunofluorescence analysis of AQP2 (red) ⁄ NeuN (green) (**C**) AQP2 (red) ⁄ CD31 (green) (**D**) and AQP2 (red) ⁄ S100 (green) expression in DRG of CCI rats. Nuclei were stained in blue. Scale bars (**A**, **B**) 10 um, (**C**-**E**) 1 um (previously published in Buffoli B *et al*., 2009 - Wiley-Blackwell).
